# Supplementary material for: Academic, clinical and personal experiences of undergraduate healthcare students during the COVID-19 pandemic: A prospective cohort study
Source: PLoS One. 2022 Jul 27;17(7):e0271873. doi: 10.1371/journal.pone.0271873 (PMC9328508; doi:10.1371/journal.pone.0271873)
Supplement: S1 File — (PDF) [file pone.0271873.s001.pdf]

An investigation of the long-term impact of the COVID-19 pandemic on the education and clinical deve

---

Start of Block: Default Question Block

Q1 What course are you registered on?

- ◯ Diagnostic Radiography and Imaging (2)
- ◯ Health Science Physiology (6)
- ◯ Occupational Therapy (5)
- ◯ Physiotherapy (4)
- ◯ Podiatry (1)
- ◯ Radiotherapy and Oncology (3)
- ◯ Speech and Language Therapy (7)

Q2 In which year of study are you currently enrolled?

- ◯ 1st year (1)
- ◯ 2nd year (2)
- ◯ 3rd year (3)

Q50 How would you describe your gender?

- Female (including transgender women) (1)
- Male (including transgender men) (2)
- Gender-fluid (4)
- Agender (5)
- Prefer not to say (6)
- Other (7) ____

Q3 What age are you?

- less than 20 years old (1)
- 20-23 years old (2)
- 24 – 30 years old (3)
- over 30 years old (4)

Q4 Click all that apply to you

- ☐ I have caring responsibilities (1)
- ☐ I have a part-time job (2)
- ☐ I have access and personal use of a car (3)
- ☐ I have a term time address that is different to my home address (4)
- ☐ I have access to reliable fast broadband (5)
- ☐ I have access to a laptop suitable for academic work (6)
- ☐ I have access to a desk suitable for academic work (7)
- ☐ I have access to a quiet/adequately sized working space (8)

Q5 Have you tested positive for COVID-19?

- ○ Yes (3)
- ○ No (4)
- ○ If you were not tested but had symptoms please give further details if applicable. If you were tested and diagnosed positive, what was this experience like? (5) ____

Q6 What are your thoughts and feelings about COVID-19?

- ☐ Thinking about the coronavirus (COVID-19) makes me feel threatened and vulnerable. (1)
- ☐ I am afraid of the coronavirus. (2)
- ☐ I am not worried about the coronavirus (3)
- ☐ I am worried that I or people I love or care about will get sick from the coronavirus (4)
- ☐ I am stressed when I am around other people because I worry I'll catch the coronavirus (5)
- ☐ I have tried hard to avoid contact with other people as I don't want to become ill. (6)
- ☐ I am not remotely concerned and happy to get on with things (7)
- ☐ I find it easy to go out as long as I adhere to with social distancing. (8)
- ☐ I feel it is all blown out of proportion and COVID-19 is just like any other flu/virus (9)

Q7 How would you rate the overall quality of the following platforms for teaching and learning?

	N/A. Have not used (1)	Excellent (2)	Average (3)	Poor (5)
Blackboard (1)	○	○	○	○
Blackboard Collaborate Ultra (2)	○	○	○	○
Panopto (3)	○	○	○	○
Recorded Lectures (4)	○	○	○	○
Blackboard Chatrooms (5)	○	○	○	○
Skype (6)	○	○	○	○
Zoom (7)	○	○	○	○
Nearpod (8)	○	○	○	○
Microsoft Teams (9)	○	○	○	○
Turning Point (10)	○	○	○	○

Q8 What is your preferred mechanism for remote teaching and learning and why? (consider the following :recorded lectures, live lectures, chat function, voice interaction, quizzes, worksheets, viewed videos, uploaded videos of self completing a task, discussion groups etc) .

____

Q9 What is your preferred mode for delivery of remote teaching and education?

- Synchronous - students and lecturers join sessions simultaneously at prearranged times. Lectures are delivered in real-time, Q&A is possible and student-lecturer and/or student-student peer interactions are possible. (1)
- Asynchronous - students access resources and listen to prerecorded lectures at a time of the student's choosing. Resources remain accessible 24/7, no lecturer is present, no live Q&A is available, students are signposted to learning resources. (2)

Q10 I prefer teaching that is...

- Consistently delivered in the University (1)
- Consistently delivered remotely (2)
- Allows some mixture of the two (3)

Q11 I am currently

- On placement (1)
- In university (2)
- Other (3)

Skip To: Q12 If I am currently = On placement

Skip To: Q15 If I am currently = In university

Skip To: Q15 If I am currently = Other

Display This Question:

If I am currently = On placement

Carry Forward Selected Choices from "I am currently "

Q12 What trust area are you working in on clinical placement?

- ○ Belfast Health and Social Care Trust (1)
- ○ Southern Health and Social Care Trust (2)
- ○ Northern Health and Social Care Trust (3)
- ○ Western Health and Social Care Trust (4)
- ○ South Eastern Health and Social Care Trust (5)
- ○ On placement (6)
- ○ In university (7)
- ○ Other (8)

Q13
How is COVID-19 impacting on your placement right now with respect to:

	Negatively (1)	No Impact (2)	Not applicable (3)	Positively (4)	Mixed (5)
Integration into the unit/department (1)	○	○	○	○	○
Development of professional skill (2)	○	○	○	○	○
Development of practical skills (4)	○	○	○	○	○
Development of interpersonal skills (6)	○	○	○	○	○
Accessibility of technology to engage with online university learning/interactions/supervision? (8)	○	○	○	○	○
Level of clinical supervision and feedback (5)	○	○	○	○	○
Opportunity to engage face to face with patients (7)	○	○	○	○	○
Assessment of your skills and knowledge (9)	○	○	○	○	○
Making and maintaining friendships (12)	○	○	○	○	○
Mental Wellbeing (14)	○	○	○	○	○
Ability to seek academic support (13)	○	○	○	○	○
Personal circumstances (10)	○	○	○	○	○
Financial circumstances (11)	○	○	○	○	○

Q14 Have you any additional comments on how COVID-19 is impacting on your placement right now?

____

Q15 How is COVID-19 impacting on the following right now move each item into the category you feel is most appropriate

Negatively	No Impact	Positively	Mixed
______ Learning environment (1)	______ Learning environment (1)	______ Learning environment (1)	______ Learning environment (1)
______ Accessibility to suitable technology (2)	______ Accessibility to suitable technology (2)	______ Accessibility to suitable technology (2)	______ Accessibility to suitable technology (2)
______ Development of practical skills (3)	______ Development of practical skills (3)	______ Development of practical skills (3)	______ Development of practical skills (3)
______ Your assessments (4)	______ Your assessments (4)	______ Your assessments (4)	______ Your assessments (4)
______ Personal circumstances (5)	______ Personal circumstances (5)	______ Personal circumstances (5)	______ Personal circumstances (5)
______ Financial circumstances (6)	______ Financial circumstances (6)	______ Financial circumstances (6)	______ Financial circumstances (6)
______ Making and maintaining friendships (7)	______ Making and maintaining friendships (7)	______ Making and maintaining friendships (7)	______ Making and maintaining friendships (7)
______ Ability to get academic support (8)	______ Ability to get academic support (8)	______ Ability to get academic support (8)	______ Ability to get academic support (8)
______ Mental Wellbeing (9)	______ Mental Wellbeing (9)	______ Mental Wellbeing (9)	______ Mental Wellbeing (9)
______ Development of your overall learning (10)	______ Development of your overall learning (10)	______ Development of your overall learning (10)	______ Development of your overall learning (10)

Q16 How comfortable/confident do you feel with face to face interactions with patients during the COVID pandemic?
0 = not confident 10 = confident

	0	1	2	3	4	5	6	7	8	9	10
1 ()											

Q17 Have you used remote consultations with patients previously (telephone reviews/ video consultations etc. on previous placements, as part of your training)?

- Yes (1)
- No (2)
- Not applicable (4)

Skip To: Q21 If Have you used remote consultations with patients previously (telephone reviews/ video consultatio... = Not applicable

Skip To: Q21 If Have you used remote consultations with patients previously (telephone reviews/ video consultatio... = No

Q18 Have you had much training/experience using remote consultations with patients?
0 = No training 10 = A lot of training

	0	1	2	3	4	5	6	7	8	9	10
Click to write Choice 1 ()											

Q19
How confident do you feel using remote consultations with patients during the COVID pandemic?0 = Not confident 10 = Confident

0	1	2	3	4	5	6	7	8	9	10

1 ()	

Q20 Can you identify any concerns you have using remote consultations with patients and how these can be addressed?

____

Q21 Regarding PPE do you feel you have:

	Yes (1)	No (2)	Unsure (3)
Adequate access to appropriate PPE (1)	○	○	○
Sufficient knowledge about appropriate PPE for your caseload (2)	○	○	○
Sufficient training, support, advice on use and donning/doffing of PPE (3)	○	○	○
Problems with wearing PPE for example allergies/irritation (4)	○	○	○

Q22 Any additional comments on PPE?

____

Q23
Has coverage of COVID-19 (news and/or social media) affected the following:

	Not at all (1)	Yes – in a positive way (2)	Yes – in a negative way (3)
Your thoughts about a career as a healthcare professional (1)	○	○	○
Decisions/thoughts about your clinical placements? (2)	○	○	○

Q24 Can you give further details on how COVID-19 has influenced your thoughts on placement and/or your choice of profession ?

____

Q25 Are you using any strategies to help keep yourself physically and mentally well at the moment?

○ Yes (1)

○ No (2)

Skip To: Q27 If Are you using any strategies to help keep yourself physically and mentally well at the moment? = No

Q26 Can you give more details on any coping strategies you are using to keep physically and mentally fit?

____

Q27 Regarding shielding self or relatives

	Yes (1)	No (2)
Do you live with someone who is shielding or at high risk? (1)	○	○
Have you altered where you would normally live to protect others? (2)	○	○

Q28
If you answered yes to shielding how did this impact on you? Financially, socially, psychologically?

____

Q29
How concerned are you about possibly transmitting/spreading COVID to a member of your household? 0 = Not concerned   10 = Very concerned

	0	1	2	3	4	5	6	7	8	9	10
1 ()											

Q30 Have you any additional comments to add regarding the impact of the COVID-19 pandemic on your learning and teaching?

____

End of Block: Default Question Block
