# Supplementary material for: Academic, clinical and personal experiences of undergraduate healthcare students during the COVID-19 pandemic: A prospective cohort study
Source: PLoS One. 2022 Jul 27;17(7):e0271873. doi: 10.1371/journal.pone.0271873 (PMC9328508; doi:10.1371/journal.pone.0271873)
Supplement: S2 File — (PDF) [file pone.0271873.s002.pdf]

Time point 2: An investigation of the long-term impact of the COVID-19 pandemic on the education and

---

Start of Block: Default Question Block

Q1 This is the same survey you answered at the start of semester but as we are learning to live with COVID-19 we would like to assess if your opinions have changed over time or remain the same. Timepoint 2: An investigation of the long-term impact of the COVID-19 pandemic on the education and clinical development

○ click to consent and continue with survey (1)

Q2 Have you completed the first version of his survey at the start of semester ?

○ Yes (1)

○ No (2)

Q3 What course are you registered on?

- ○ Diagnostic Radiography and Imaging (2)
- ○ Healthcare Science/Health Physiology (6)
- ○ Occupational Therapy (5)
- ○ Physiotherapy (4)
- ○ Podiatry (1)
- ○ Radiotherapy and Oncology (3)
- ○ Speech and Language Therapy (7)

Q4 In which year of study are you currently enrolled?

- ○ 1st year (1)
- ○ 2nd year (2)
- ○ 3rd year (3)

Q5 How would you describe your gender?

- ○ Female (including transgender women) (1)
- ○ Male (including transgender men) (2)
- ○ Gender-fluid (4)
- ○ Agender (5)
- ○ Prefer not to say (6)
- ○ Other (7) ____

Q6 What age are you?

- less than 20 years old (1)
- 20-23 years old (2)
- 24 – 30 years old (3)
- over 30 years old (4)

Q7 Click all that apply to you

- I have caring responsibilities (1)
- I have a part-time job (2)
- I have access and personal use of a car (3)
- I have a term time address that is different to my home address (4)
- I have access to reliable fast broadband (5)
- I have access to a laptop suitable for academic work (6)
- I have access to a desk suitable for academic work (7)
- I have access to a quiet/adequately sized working space (8)

Q8 Have you tested positive for COVID-19?

- ◯ Yes (3)
- ◯ No (4)
- ◯ If you were not tested but had symptoms please give further details if applicable. If you were tested and diagnosed positive, what was this experience like? (5) ____

Q9 Have you had to self-isolate because of close contact ?

- ◯ Yes at the start of the pandemic (1)
- ◯ Yes since the start of semester 1 (4)
- ◯ No I have not had to self-isolate (6)

Skip To: Q13 If Have you had to self-isolate because of close contact ? = No I have not had to self-isolate

---

Q10 If you had to self-isolate, how did you know ?

- ◯ I had symptoms (1)
- ◯ I had symptoms and a positive test for COVID-19 (2)
- ◯ I was notified through a friend/family/work colleague/flat mate (3)
- ◯ Notified through phone app (4)

Q11 Has this impacted on your learning and teaching ?

- ○ Yes I missed practicals/classes in university (1)
- ○ Yes I missed clinical placement (2)
- ○ I fell behind with my study (4)
- ○ No it did not impact on me (5)

Q12 Were you allowed the opportunity to catch up on what you missed (if anything)…please give details

____

Q13 What are your thoughts and feelings about COVID-19?

- ☐ Thinking about the coronavirus (COVID-19) makes me feel threatened and vulnerable. (1)
- ☐ I am afraid of the coronavirus. (2)
- ☐ I am not worried about the coronavirus (3)
- ☐ I am worried that I or people I love or care about will get sick from the coronavirus (4)
- ☐ I am stressed when I am around other people because I worry I'll catch the coronavirus (5)
- ☐ I have tried hard to avoid contact with other people as I don't want to become ill. (6)
- ☐ I am not remotely concerned and happy to get on with things (7)
- ☐ I find it easy to go out as long as I adhere to with social distancing. (8)
- ☐ I feel it is all blown out of proportion and COVID-19 is just like any other flu/virus (9)

Q14 How would you rate the overall quality of the following platforms for teaching and learning?

	N/A. Have not used (1)	Excellent (2)	Average (3)	Poor (5)
Blackboard (1)	○	○	○	○
Blackboard Collaborate Ultra (2)	○	○	○	○
Panopto (3)	○	○	○	○
Recorded Lectures (4)	○	○	○	○
Blackboard Chatrooms (5)	○	○	○	○
Skype (6)	○	○	○	○
Zoom (7)	○	○	○	○
Nearpod (8)	○	○	○	○
Microsoft Teams (9)	○	○	○	○
Turning Point (10)	○	○	○	○

Q15 What is your preferred mechanism for remote teaching and learning and why? (consider the following :recorded lectures, live lectures, chat function, voice interaction, quizzes, worksheets, viewed videos, uploaded videos of self completing a task, discussion groups etc) .

____

Q16 What is your preferred mode for delivery of remote teaching and education?

- ○ Synchronous - students and lecturers join sessions simultaneously at prearranged times. Lectures are delivered in real-time, Q&A is possible and student-lecturer and/or student-student peer interactions are possible. (1)
- ○ Asynchronous - students access resources and listen to prerecorded lectures at a time of the student's choosing. Resources remain accessible 24/7, no lecturer is present, no live Q&A is available, students are signposted to learning resources. (2)

Q17 I prefer teaching that is...

- ○ Consistently delivered in the University (1)
- ○ Consistently delivered remotely (2)
- ○ Allows some mixture of the two (3)

Q18 Have you been on campus for lectures since semester 1 started?

- ○ yes practicals (1)
- ○ yes seminars/teaching (2)
- ○ Yes other (3)
- ○ No I have worked remotely (4)

Q19 Were you happy to come onsite or have you any comments you would like to make?

____

Q20 Have your opinions of COVID-19 changed since the start of this semester?

- ◯ I feel more stressed about the pandemic now that I am back at university (1)
- ◯ I feel more stressed about the pandemic because I am on clinical placement (2)
- ◯ My feelings haven't changed (3)
- ◯ I feel less stressed about the pandemic (4)
- ◯ I am coping better and learning to live with the pandemic (5)

Q21 I am currently

- ▢ On placement (1)
- ▢ In university (2)
- ▢ Other (3)

Skip To: Q22 If I am currently = On placement
Skip To: Q25 If I am currently = In university
Skip To: Q25 If I am currently = Other

Display This Question:
If I am currently = On placement

Carry Forward Selected Choices from "I am currently "

X→

Q22 What trust area are you working in on clinical placement?

- ○ Belfast Health and Social Care Trust (1)
- ○ Southern Health and Social Care Trust (2)
- ○ Northern Health and Social Care Trust (3)
- ○ Western Health and Social Care Trust (4)
- ○ South Eastern Health and Social Care Trust (5)
- ○ On placement (6)
- ○ In university (7)
- ○ Other (8)

Q23
How is COVID-19 impacting on your placement right now with respect to:

	Negatively (1)	No Impact (2)	Not applicable (3)	Positively (4)	Mixed (5)
Integration into the unit/department (1)	○	○	○	○	○
Development of professional skill (2)	○	○	○	○	○
Development of practical skills (4)	○	○	○	○	○
Development of interpersonal skills (6)	○	○	○	○	○
Accessibility of technology to engage with online university learning/interactions/supervision? (8)	○	○	○	○	○
Level of clinical supervision and feedback (5)	○	○	○	○	○
Opportunity to engage face to face with patients (7)	○	○	○	○	○
Assessment of your skills and knowledge (9)	○	○	○	○	○
Making and maintaining friendships (12)	○	○	○	○	○
Mental Wellbeing (14)	○	○	○	○	○
Ability to seek academic support (13)	○	○	○	○	○
Personal circumstances (10)	○	○	○	○	○
Financial circumstances (11)	○	○	○	○	○

Q24 Have you any additional comments on how COVID-19 is impacting on your placement right now?

____

Q25 How is COVID-19 impacting on the following right now move each item into the category you feel is most appropriate

Negatively	No Impact	Positively	Mixed
______ Learning environment (1)	______ Learning environment (1)	______ Learning environment (1)	______ Learning environment (1)
______ Accessibility to suitable technology (2)	______ Accessibility to suitable technology (2)	______ Accessibility to suitable technology (2)	______ Accessibility to suitable technology (2)
______ Development of practical skills (3)	______ Development of practical skills (3)	______ Development of practical skills (3)	______ Development of practical skills (3)
______ Your assessments (4)	______ Your assessments (4)	______ Your assessments (4)	______ Your assessments (4)
______ Personal circumstances (5)	______ Personal circumstances (5)	______ Personal circumstances (5)	______ Personal circumstances (5)
______ Financial circumstances (6)	______ Financial circumstances (6)	______ Financial circumstances (6)	______ Financial circumstances (6)
______ Making and maintaining friendships (7)	______ Making and maintaining friendships (7)	______ Making and maintaining friendships (7)	______ Making and maintaining friendships (7)
______ Ability to get academic support (8)	______ Ability to get academic support (8)	______ Ability to get academic support (8)	______ Ability to get academic support (8)
______ Mental Wellbeing (9)	______ Mental Wellbeing (9)	______ Mental Wellbeing (9)	______ Mental Wellbeing (9)
______ Development of your overall learning (10)	______ Development of your overall learning (10)	______ Development of your overall learning (10)	______ Development of your overall learning (10)

Q26 How comfortable/confident do you feel with face to face interactions with patients during the COVID pandemic?
0 = not confident   10 = confident

	0	1	2	3	4	5	6	7	8	9	10
1 ()											

Q27 Have you used remote consultations with patients previously (telephone reviews/ video consultations etc. on previous placements, as part of your training)?

- Yes (1)
- No (2)
- Not applicable (4)

Skip To: Q31 If Have you used remote consultations with patients previously (telephone reviews/ video consultatio... = Not applicable

Skip To: Q31 If Have you used remote consultations with patients previously (telephone reviews/ video consultatio... = No

Q28 Have you had much training/experience using remote consultations with patients?
0 = No training   10 = A lot of training

	0	1	2	3	4	5	6	7	8	9	10
Click to write Choice 1 ()											

Q29
How confident do you feel using remote consultations with patients during the COVID pandemic?0 = Not confident   10 = Confident

0	1	2	3	4	5	6	7	8	9	10

1 ()	

Q30 Can you identify any concerns you have using remote consultations with patients and how these can be addressed?

____

Q31 Regarding PPE do you feel you have:

	Yes (1)	No (2)	Unsure (3)
Adequate access to appropriate PPE (1)	○	○	○
Sufficient knowledge about appropriate PPE for your caseload (2)	○	○	○
Sufficient training, support, advice on use and donning/doffing of PPE (3)	○	○	○
Problems with wearing PPE for example allergies/irritation (4)	○	○	○
Problems with FIT testing of masks (11)	○	○	○

Q32 Any additional comments on PPE?

____

Q33
Has coverage of COVID-19 (news and/or social media) affected the following:

	Not at all (1)	Yes – in a positive way (2)	Yes – in a negative way (3)
Your thoughts about a career as a healthcare professional (1)	○	○	○
Decisions/thoughts about your clinical placements? (2)	○	○	○

Q34 Can you give further details on how COVID-19 has influenced your thoughts on placement and/or your choice of profession ?

____

Q35 Are you using any strategies to help keep yourself physically and mentally well at the moment?

○ Yes (1)

○ No (2)

Skip To: Q37 If Are you using any strategies to help keep yourself physically and mentally well at the moment? = No

Q36 Can you give more details on any coping strategies you are using to keep physically and mentally fit?

____

Q37 Where did you live during semester 1?

- ◯ term time address (1)
- ◯ at hospital accommodation (2)
- ◯ university halls of residence (3)
- ◯ at home (4)
- ◯ started in one of the first 3 but moved home as semester progressed (5)

Skip To: Q40 If Where did you live during semester 1? = at home

---

Q38 If you moved home were you able to terminate your contract on your term time address ?

- ◯ Yes (1)
- ◯ No (2)
- ◯ Negotiating it at present (3)
- ◯ Not applicable (4)

Q39 If you didn’t move home, why did you stay in your term time address if lectures were online?

____

Q40 Regarding shielding self or relatives

	Yes (1)	No (2)
Do you live with someone who is shielding or at high risk? (1)	○	○
Have you altered where you would normally live to protect others? (2)	○	○

Q41
If you answered yes to shielding how did this impact on you? Financially, socially, psychologically?

____

Q42
How concerned are you about possibly transmitting/spreading COVID to a member of your household? 0 = Not concerned 10 = Very concerned

	0	1	2	3	4	5	6	7	8	9	10
1 ()											

Q43 Have you any additional comments to add regarding the impact of the COVID-19 pandemic on your learning and teaching?

____

Q44 We would really like to explore the results of this survey further. If you would like to participate in a focus group in semester two please insert your email address in the box below.

____

Q45 If you would like to participate in a focus group in semester two please tick which date suits.

- ◯ Focus group 1- 26th of January 19:00 (Tues) (1)
- ◯ Focus group 2- 15th of February 19:00 (Mon) (4)
- ◯ Focus group 3 - 9th of March 19:00 (Tues) (5)

Q46 Thank you for taking the time to complete this survey. It is very much appreciated.

End of Block: Default Question Block
