# Supplementary material for: Academic, clinical and personal experiences of undergraduate healthcare students during the COVID-19 pandemic: A prospective cohort study
Source: PLoS One. 2022 Jul 27;17(7):e0271873. doi: 10.1371/journal.pone.0271873 (PMC9328508; doi:10.1371/journal.pone.0271873)
Supplement: S3 File — (PDF) [file pone.0271873.s003.pdf]

T3 sem2: An investigation of the long-term impact of the COVID-19 pandemic on the students

Start of Block: Default Question Block

Q1 This is an amended version of the survey you answered last semester. As we are learning to live with COVID-19 and using feedback from our focus groups we would like to know your opinions on a few specific areas.
Timepoint 3: An investigation of the long-term impact of the COVID-19 pandemic on the education and clinical development

○ click to consent and continue with survey (1)

Page Break

Q2 Have you completed the first version of this survey at the start of semester 1?

- ○ Yes (1)
- ○ No (2)

Q66 Have you completed the second version of this survey at the end of semester 1?

- ○ Yes (21)
- ○ No (22)

Q3 What course are you registered on?

- ○ Diagnostic Radiography and Imaging (2)
- ○ Healthcare Science/Health Physiology (6)
- ○ Occupational Therapy (5)
- ○ Physiotherapy (4)
- ○ Podiatry (1)
- ○ Radiotherapy and Oncology (3)
- ○ Speech and Language Therapy (7)

Q4 In which year of study are you currently enrolled?

- ○ 1st year (1)
- ○ 2nd year (2)
- ○ 3rd year (3)

Q5 How would you describe your gender?

- ○ Female (including transgender women) (1)
- ○ Male (including transgender men) (2)
- ○ Gender-fluid (4)
- ○ Agender (5)
- ○ Prefer not to say (6)
- ○ Other (7) ____

Q6 What age are you?

- ○ less than 20 years old (1)
- ○ 20-23 years old (2)
- ○ 24 – 30 years old (3)
- ○ over 30 years old (4)

Page Break

Q7 Click all that apply to you

- [ ] I have caring responsibilities (1)
- [ ] I have a part-time job (2)
- [ ] I have access and personal use of a car (3)
- [ ] I have a term time address that is different to my home address (4)
- [ ] I have access to reliable fast broadband (5)
- [ ] I have access to a laptop suitable for academic work (6)
- [ ] I have access to a desk suitable for academic work (7)
- [ ] I have access to a quiet/adequately sized working space (8)

Q67 How confident do you feel using a computer? 0 = not confident 10 = confident

	0	10
Click to write Choice 1 ()		

Q68 Has your computer literacy impacted your ability to learn online using remote delivery ?

- ○ Yes (21)
- ○ No (22)

Q69 If your computer literacy has impacted your ability to learn online using remote delivery, can you give further details please?

____

Page Break

Q8 Have you tested positive for COVID-19?

- ○ Yes (3)
- ○ No (4)
- ○ If you were not tested but had symptoms please give further details if applicable. If you were tested and diagnosed positive, what was this experience like? (5) ____

Q9 Have you had to self-isolate because of close contact ?

- ○ Yes at the start of the pandemic (1)
- ○ Yes since the start of semester 1 (4)
- ○ No I have not had to self-isolate (6)

Skip To: Q13 If Have you had to self-isolate because of close contact ? = No I have not had to self-isolate

Q10 If you had to self-isolate, how did you know ?

- ▢ I had symptoms (1)
- ▢ I had symptoms and a positive test for COVID-19 (2)
- ▢ I was notified through a friend/family/work colleague/flat mate (3)
- ▢ Notified through HSC Test and Trace app on phone (4)

Q11 Has this impacted on your learning and teaching ?

- ☐ Yes I missed practicals/classes in university (1)
- ☐ Yes I missed clinical placement (2)
- ☐ I fell behind with my study (4)
- ☐ No it did not impact on me (5)

Q12 Were you allowed the opportunity to catch up on what you missed (if anything)…please give details

____

Page Break

Q13 What are your thoughts and feelings about COVID-19 after living with it for a year? (Please tick all that apply)

- ☐ I am not ready to return to normal working and teaching in classrooms until I get vaccinated (1)
- ☐ I am afraid of the coronavirus. (2)
- ☐ I do not want to return to university for classes and want to continue with online learning full time (3)
- ☐ I am so fed up living a socially distanced lifestyle and I want to get back to normal (4)
- ☐ I am afraid this lockdown and social distancing is not going to end. (5)
- ☐ I am worried I will feel awkward in crowds and lecture rooms when the lockdown ends (6)
- ☐ I am worried that I have lost touch with my friends, classmates and work colleagues (11)
- ☐ I am concerned about the amount of time I am sitting at a computer staring at a monitor (7)
- ☐ I find it easy to social distance and this working/teaching style suits me and my needs (8)
- ☐ I am happy to return to university for classes but would rather continue with online learning full time (9)
- ☐ I am happy to return to university for classes but would like some online learning to be maintained in the course delivery (12)
- ☐ I have managed to control my computer screen time and manage regular breaks throughout the day (13)

Q15 What is your preferred mechanism for remote teaching and learning and why? (consider the following :recorded lectures, live lectures, chat function, voice interaction, quizzes, worksheets, viewed videos, uploaded videos of self completing a task, discussion groups etc) . Please list the top 3 in order of preference

____

Q70 Did you use breakout rooms or perform groupwork using any of the teaching and learning platforms?

- ○ Yes (21)
- ○ No (22)

Q71 Did this groupwork or use of breakout rooms aid you to form/maintain friendships during remote delivery?

- ○ Yes (21)
- ○ No (22)

Q72 If groupwork or breakout rooms helped you to form/maintain friendships during remote delivery can you give further details on how?

____

Q73 Did groupwork or breakout rooms aid you to learn and work on tasks during remote delivery

- ○ Yes (21)
- ○ No (22)

Q74 Can you give more details on how breakout rooms helped you to learn and work on tasks during remote delivery?

____

Q75 Did you find it easy to engage with your peers during the breakout rooms?

- Yes (21)
- No (22)

Q76 Can you give further details of your experience engaging with your peers during the breakout rooms?

____

Q77 During lecture delivery were you able to ask questions if you were unsure of any material?

- Yes (21)
- No (22)

Q78 Can you give more details on your experience of questions and feedback from academic staff during lecture delivery.

____

Q79 Would you prefer for all participants to have their cameras on and be visible during online lectures?

- ○ Yes (21)
- ○ No (22)

Q80 Overall, do you feel remote delivery has impacted your ability to get a response from academic staff regarding your course queries ?

- ○ Yes (26)
- ○ No (27)

Q81 If yes please clarify how remote delivery has impacted your ability to get a response from academic staff regarding your course queries.

____

Q82 Did online delivery affect your assessments?

- ▢ Our assessment schedule was unchanged (1)
- ▢ Assessments were delivered online remotely (2)
- ▢ Assessments were moved from semester 1 to semester 2 and added to our workload (3)
- ▢ Assessments have been delayed and are not completed yet (4)

Q16 What is your preferred mode for delivery of remote teaching and education?

○ Synchronous - students and lecturers join sessions simultaneously at prearranged times. Lectures are delivered in real-time, Q&A is possible and student-lecturer and/or student-student peer interactions are possible. (1)

○ Asynchronous - students access resources and listen to prerecorded lectures at a time of the student's choosing. Resources remain accessible 24/7, no lecturer is present, no live Q&A is available, students are signposted to learning resources. (2)

○ Mixed delivery of Synchronous and Asynchronous (4)

Q17 I prefer teaching that is...

○ Consistently delivered in the University (1)

○ Consistently delivered remotely (2)

○ Allows some mixture of the two (3)

Q18 Have you been on campus since semester 2 started?

▢ yes practicals (1)

▢ yes seminars/teaching (2)

▢ Yes other (3)

▢ No I have worked remotely (4)

Q19 Were you happy to come onsite or have you any comments you would like to make?

____

Q84 What impact has remote learning had on your peer learning? (Please tick all that apply)

- [ ] I am missing out on peer learning with my class mates. We always discussed/ clarified material whilst travelling/car sharing en route to university. (1)
- [ ] I am missing out on peer learning with my class mates as we always discussed/clarified material whilst having a coffee/tea/chat. (2)
- [ ] I am missing out on peer learning with my class mates as we always discussed/clarified material whilst exercising. (3)
- [ ] I never really discuss lectures with classmates so I don't feel I'm missing anything. (4)
- [ ] I prefer to learn by myself (5)

Q20 Have your opinions of COVID-19 changed since the start of this semester?

- [ ] I feel more stressed about the pandemic now that I am back at university (1)
- [ ] I feel more stressed about the pandemic because I am on clinical placement (2)
- [ ] My feelings haven't changed (3)
- [ ] I feel less stressed about the pandemic (4)
- [ ] I am coping better and learning to live with the pandemic (5)

Q85 Have you additional comments regarding the workload distribution across semester 1 and 2. In your opinion was this evenly distributed?

____

Page Break

Q21 I am currently

- ☐ On placement (1)
- ☐ In university (2)
- ☐ Other (3)

Skip To: Q22 If I am currently = On placement
Skip To: Q33 If I am currently = In university
Skip To: Q33 If I am currently = Other

Display This Question:
If I am currently = On placement
Carry Forward Selected Choices from "I am currently "

Q22 What trust area are you working in on clinical placement?

- ○ Belfast Health and Social Care Trust (1)
- ○ Southern Health and Social Care Trust (2)
- ○ Northern Health and Social Care Trust (3)
- ○ Western Health and Social Care Trust (4)
- ○ South Eastern Health and Social Care Trust (5)
- ○ On placement (6)
- ○ In university (7)
- ○ Other (8)

Q86 What are your thoughts about completing clinical placement during a pandemic? (Please tick all that apply)

- ☐ I did not feel prepared to go into placement (1)
- ☐ I do not think students should have to go on clinical placement during a pandemic. Placement should be postponed until it is over. (2)
- ☐ There was a limited range of work in the hospital/community as clinics were cancelled to accommodate COVID-19 patients mainly. (3)
- ☐ I was limited in the places/departments/rooms I could attend due to social distancing measures. (4)
- ☐ I feel my confidence has decreased as I didn’t get enough hands on experience (5)
- ☐ I feel I was stigmatised as I was a “student”. People thought I was one of the rule breakers who wasn’t socially distancing outside work and hence a risk to others in work.Click to write Choice 6 (6)
- ☐ I had to leave my part-time job as colleagues thought I was at higher risk of catching COVID-19 working in the hospital. (7)
- ☐ I had to leave my part-time job as I was at risk of catching COVID-19 from my co-workers and then bring it into the hospital staff/patients. (8)
- ☐ I feel the university fully prepared me for placement (9)
- ☐ I feel my confidence has increased as I got to see very difficult and challenging cases with lots of hands on experience (10)
- ☐ I was not restricted in my day to day working and I got to see everything my colleagues were doing. (11)
- ☐ I found placement during a pandemic very rewarding and learned how to cope in difficult circumstances. (12)

Q23
How is COVID-19 impacting on your placement right now with respect to:

	Negatively (1)	No Impact (2)	Not applicable (3)	Positively (4)	Mixed (5)
Integration into the unit/department (1)	○	○	○	○	○
Development of professional skill (2)	○	○	○	○	○
Development of practical skills (4)	○	○	○	○	○
Development of interpersonal skills (6)	○	○	○	○	○
Accessibility of technology to engage with online university learning/interactions/supervision? (8)	○	○	○	○	○
Level of clinical supervision and feedback (5)	○	○	○	○	○
Opportunity to engage face to face with patients (7)	○	○	○	○	○
Assessment of your skills and knowledge (9)	○	○	○	○	○
Making and maintaining friendships (12)	○	○	○	○	○
Mental Wellbeing (14)	○	○	○	○	○
Ability to seek academic support (13)	○	○	○	○	○
Personal circumstances (10)	○	○	○	○	○
Financial circumstances (11)	○	○	○	○	○

Q24 Have you any additional comments on how COVID-19 is impacting on your placement right now?

____

Q26 How comfortable/confident do you feel with face to face interactions with patients during the COVID pandemic?
0 = not confident   10 = confident

	0	1	2	3	4	5	6	7	8	9	10
1 ()											

Q27 Have you used remote consultations with patients previously (telephone reviews/ video consultations etc. on previous placements, as part of your training)?

○ Yes (1)

○ No (2)

○ Not applicable (4)

Skip To: Q31 If Have you used remote consultations with patients previously (telephone reviews/ video consultatio... = Not applicable

Skip To: Q31 If Have you used remote consultations with patients previously (telephone reviews/ video consultatio... = No

Q28 Have you had much training/experience using remote consultations with patients?
0 = No training   10 = A lot of training

	0	1	2	3	4	5	6	7	8	9	10
Click to write Choice 1 ()											

Q29
How confident do you feel using remote consultations with patients during the COVID pandemic?0 = Not confident 10 = Confident

	0	1	2	3	4	5	6	7	8	9	10
1 ()											

Q30 Can you identify any concerns you have using remote consultations with patients and how these can be addressed?

____

Q31 Regarding PPE do you feel you have:

	Yes (1)	No (2)	Unsure (3)
Adequate access to appropriate PPE (1)	○	○	○
Sufficient knowledge about appropriate PPE for your caseload (2)	○	○	○
Sufficient training, support, advice on use and donning/doffing of PPE (3)	○	○	○
Problems with wearing PPE for example allergies/irritation (4)	○	○	○
Problems with FIT testing of masks (11)	○	○	○

Q32 Any additional comments on PPE?

____

Q33
Has coverage of COVID-19 (news and/or social media) affected the following:

	Not at all (1)	Yes – in a positive way (2)	Yes – in a negative way (3)
Your thoughts about a career as a healthcare professional (1)	○	○	○
Decisions/thoughts about your clinical placements? (2)	○	○	○

Q34 Can you give further details on how COVID-19 has influenced your thoughts on placement and/or your choice of profession ?

____

Q35 Are you using any strategies to help keep yourself physically and mentally well at the moment?

○ Yes (1)

○ No (2)

Skip To: Q37 If Are you using any strategies to help keep yourself physically and mentally well at the moment? = No

Q36 Can you give more details on any coping strategies you are using to keep physically and mentally fit?

____

Q37 Where did you live during semester 2?

- ○ term time address (1)
- ○ at hospital accommodation (2)
- ○ university halls of residence (3)
- ○ at home (4)
- ○ started in one of the first 3 but moved home as semester progressed (5)

Skip To: Q40 If Where did you live during semester 2? = at home

Q38 If you moved home were you able to terminate your contract on your term time address ?

- ○ Yes (1)
- ○ No (2)
- ○ Negotiating it at present (3)
- ○ Not applicable (4)

Q39 If you didn’t move home, why did you stay in your term time address if lectures were online?

____

Q40 Regarding shielding self or relatives

	Yes (1)	No (2)
Do you live with someone who is shielding or at high risk? (1)	○	○
Have you altered where you would normally live to protect others? (2)	○	○

Q41
If you answered yes to shielding how did this impact on you? Financially, socially, psychologically?

____

Q42
How concerned are you about possibly transmitting/spreading COVID to a member of your household? 0 = Not concerned   10 = Very concerned

	0 1 2 3 4 5 6 7 8 9 10
1 ()	

Q87 What are your thoughts and feelings about the vaccine? (Please tick all that apply)

- ☐ I have already got it (1)
- ☐ I will get it as soon as possible (2)
- ☐ I will take the vaccine but I am hesitant and apprehensive about it (3)
- ☐ I am in no hurry to get it (4)
- ☐ I will wait until I see what the side effects are (5)
- ☐ I will wait until I can get the second dose within the time frame recommended by the manufacturers (6)
- ☐ I do not want the vaccine as I am concerned at how quickly it was produced and approved (7)
- ☐ I am afraid that people will be less cautious once they get the vaccine. (8)
- ☐ I think COVID-19 is like the cold/flu and I do not need a vaccine. (9)

Page Break

Q88 Have you any additional thoughts regarding the vaccine for COVID-19 ?

____

Q89 What have you taken from the COVID-19 experience and its impact on your teaching and learning?

- ☐ I think the whole experience has had a negative impact on me (1)
- ☐ I have been given the opportunity to complete a research placement as part of my course (2)
- ☐ I have developed new skills (clinically, academically, professionally, personally) (3)
- ☐ I think the whole experience has had a positive impact on me (4)

Q43 Have you any additional comments to add regarding the impact of the COVID-19 pandemic on your learning and teaching?

____

Q46 Thank you for taking the time to complete this survey. It is very much appreciated.

End of Block: Default Question Block

Start of Block: Block 1
