# Supplementary material for: Academic, clinical and personal experiences of undergraduate healthcare students during the COVID-19 pandemic: A prospective cohort study
Source: PLoS One. 2022 Jul 27;17(7):e0271873. doi: 10.1371/journal.pone.0271873 (PMC9328508; doi:10.1371/journal.pone.0271873)
Supplement: Attachment — Submitted filename: Response to Reviewers R1.docx [file pone.0271873.s004.docx]

**Response to Reviewers**

**Reference :** PONE-D-21-29579R1

**Original Title :** The impact of the COVID-19 pandemic on healthcare students.

**Submitted to :** PLOS ONE

**Due : 20** th April 2022

To Whom it may concern.

Please see rebuttal information below in response to each point raised by the academic editor and reviewers.

1. Please amend the title either on the online submission form or in your manuscript so that they are identical. This has been amended accordingly.

2. Please provide additional details regarding participant consent. In the Methods section, please ensure that you have specified (1) whether consent was informed and (2) what type you obtained (for instance, written or verbal). If your study included minors, state whether you obtained consent from parents or guardians. If the need for consent was waived by the ethics committee, please include this information. Additional details have been added regarding informed participant consent gained electronically. No minors were included as these were university students.
