# Supplementary material for: Academic, clinical and personal experiences of undergraduate healthcare students during the COVID-19 pandemic: A prospective cohort study
Source: PLoS One. 2022 Jul 27;17(7):e0271873. doi: 10.1371/journal.pone.0271873 (PMC9328508; doi:10.1371/journal.pone.0271873)
Supplement: Attachment — Submitted filename: Response to Reviewers 26.4.22.docx [file pone.0271873.s005.docx]

**Response to Reviewers**

**Reference :** PONE-D-21-29579R1

**Original Title :** The impact of the COVID-19 pandemic on healthcare students.

**Submitted to :** PLOS ONE

**Due :** 27^th^ May 2022

To Whom it may concern.

Please see rebuttal information below in response to the Additional Editor Comments.

A lot of medical research is based on observation and to avoid observational studies' reporting to be compromised for the quality, we recommend using STROBE checklist.
Please ensure the reporting of your observational study using the STROBE checklist ([http://www.strobe-statement.org](https://eur03.safelinks.protection.outlook.com/?url=http%3A%2F%2Fwww.strobe-statement.org%2F&data=04%7C01%7CGuille-S1%40ulster.ac.uk%7C25f1edba7153419d575f08da1ed3c808%7C6f0b94874fa842a8aeb4bf2e2c22d4e8%7C0%7C0%7C637856192185335536%7CUnknown%7CTWFpbGZsb3d8eyJWIjoiMC4wLjAwMDAiLCJQIjoiV2luMzIiLCJBTiI6Ik1haWwiLCJXVCI6Mn0%3D%7C3000&sdata=cG9%2FH1N%2FLhYruVyBVi%2FlT%2FiErD2BeWXTwjH9etAigqY%3D&reserved=0)). The STROBE Statement consists of a checklist of 22 items, which relate to the title, abstract, introduction, methods, results, and discussion sections of articles.

Strobe checklist reviewed in line with manuscript.

Under Discussion, abbreviated terms 'TLEs and VLEs' are used without being spelled in full elsewhere. Please correct.

TEL and VLEs already spelled in full in the “Introduction” section see line 86 therefore manuscript not changed.
